# Efficacy and safety of pulsed radiofrequency combined with nerve block for cervical radiculopathy: a systematic review and meta-analysis

**DOI:** 10.3389/fneur.2026.1877201

**Published:** 2026-09-18

**Authors:** Xiaogang Hao, Xinxin Tian, Fengling Sun, Qi An, Jiajun Zhao, Yueliang Sun, Guohui Zhou, Mingpeng Shi, Xinhua Chen

**Affiliations:** 1 College of Acupuncture and Tuina, Changchun University of Chinese Mdicine, Changchun, China; 2 Department of Pain Management, The Affiliated Hospital of Changchun University of Chinese Medicine, Changchun, China; 3 College of Traditional Chinese Medicine, Changchun University of Chinese Medicine, Changchun, China; 4 Orthopedic Department, The Affiliated Hospital of Changchun University of Chinese Medicine, Changchun, China; 5 Department of Acupuncture, The Affiliated Hospital of Changchun University of Chinese Medicine, Changchun, China

**Keywords:** cervical radiculopathy, meta-analysis, nerve block, pain management, pulsed radiofrequency

## Abstract

**Background:**

Cervical radiculopathy is a common cause of neck–shoulder–arm pain and functional impairment. Although nerve block provides rapid analgesia, its durability may be limited. This systematic review and meta-analysis evaluated whether pulsed radiofrequency combined with nerve block offers superior efficacy and comparable safety to nerve block alone.

**Methods:**

Eight databases were searched from inception to April 10, 2026. Randomized controlled trials comparing pulsed radiofrequency plus nerve block with nerve block alone in adults with cervical radiculopathy or cervical nerve root pain were included. Meta-analysis was performed using RevMan 5.4. Risk ratios or mean differences with 95% confidence intervals were calculated.

**Results:**

Nine randomized controlled trials involving 783 patients were included. Compared with nerve block alone, combined therapy significantly improved the overall clinical response rate (RR = 1.13, 95% CI: 1.05–1.22), reduced pain intensity measured by VAS score (MD = −0.87, 95% CI: −1.27 to −0.47), increased cervical flexion–extension range of motion (MD = 8.56, 95% CI: 1.67–15.44), and improved Yasuhisa Tanaka symptom scores (MD = 2.61, 95% CI: 1.81–3.40). No significant difference was observed in adverse reactions (RR = 0.96, 95% CI: 0.52–1.78).

**Discussion:**

Pulsed radiofrequency combined with nerve block may provide greater pain relief and better overall clinical response than nerve block alone without increasing short-term adverse reactions. Nevertheless, the evidence for functional improvement remains uncertain. The pooled pain difference was modest, functional outcomes were supported by few studies, and the available evidence does not establish durable benefit beyond the reported follow-up periods. Further high-quality multicenter trials are needed to confirm long-term efficacy, clarify functional benefits, and optimize treatment protocols.

**Systematic review registration:**

https://www.crd.york.ac.uk/PROSPERO/view/CRD420251271276, Identifier: CRD420251271276.

## 1 Introduction

Neck–shoulder–arm pain is not merely a localized pain problem, but represents a substantial neuromusculoskeletal disease burden that affects work capacity, sleep quality, and daily function. Among its causes, cervical radiculopathy and cervical nerve root pain are often induced by cervical nerve root compression, inflammatory irritation, or both, and are manifested as dermatomal pain, numbness, paresthesia, and even restricted motor function (1–3). With population aging, prolonged desk-based work, and increased detection rates on imaging, establishing a safer, more durable, and generalizable stepwise minimally invasive treatment strategy across pharmacological therapy, rehabilitation, and surgery has become a shared concern in pain medicine, spine surgery, and rehabilitation medicine (3, 4).

The pathological basis of cervical radiculopathy is not limited to mechanical compression, but also involves multiple mechanisms, including nerve root edema, local release of inflammatory mediators, abnormal discharge of the dorsal root ganglion, and peripheral and central sensitization (1, 3, 5). Selective nerve root block or epidural steroid injection may reduce inflammatory responses and block nociceptive afferent input through local anesthetics and glucocorticoids, thereby achieving short-term pain relief and functional improvement in some patients; however, uncertainties remain regarding the duration of efficacy, the need for repeated injections, and long-term benefits (4, 6, 7). Pulsed radiofrequency (PRF) is a non-destructive neuromodulation technique that is typically applied to the dorsal root ganglion or the affected nerve root within a temperature range that does not induce thermocoagulative injury, and it may alleviate chronic radicular pain by modulating abnormal neural discharge, neuroimmune responses, and the expression of inflammatory factors (5, 8, 9). Previous randomized trials and meta-analyses suggest that PRF can improve pain outcomes in patients with cervical radicular pain and may maintain its analgesic effect over longer follow-up periods (8, 10, 11).

Although selective nerve root block is widely used for cervical radicular pain, its effect is mainly associated with rapid anti-inflammatory analgesia, while the durability of pain relief, the need for repeated injections, and the long-term functional benefit remain uncertain (3, 10, 12). PRF, by contrast, may provide sustained neuromodulation and has shown potential benefits in cervical radicular pain, but previous evidence has mostly evaluated PRF or nerve block separately rather than their combined use (10, 11). In clinical practice, PRF combined with nerve block is often considered for patients who respond poorly to conservative treatment but do not yet have clear surgical indications; however, the available studies differ in PRF parameters, block targets, imaging guidance, follow-up duration, and outcome measures (11, 13). These inconsistencies make it difficult for clinicians to determine whether combination therapy truly offers additional benefit over nerve block alone. Therefore, a systematic review and meta-analysis is needed to clarify the efficacy, safety, and functional value of PRF combined with nerve block for cervical radiculopathy.

Therefore, this study aims to systematically evaluate, through a meta-analysis, the efficacy and safety of pulsed radiofrequency combined with nerve block for the treatment of cervical radiculopathy or cervical radicular pain, and to directly compare this approach with nerve block alone. This study focuses on clinically relevant outcomes, including pain scores, overall clinical response rate, cervical function or range of motion, symptom scores, and the incidence of adverse events, in order to test the hypothesis that pulsed radiofrequency combined with nerve block may provide more comprehensive pain control and functional benefits than nerve block alone without increasing the short-term risk of adverse reactions. The purpose of this study is not merely to confirm whether a given interventional technique is effective, but to provide a more interpretable evidence base that more closely reflects clinical decision-making scenarios for stepwise and individualized minimally invasive treatment in patients with refractory cervical radiculopathy.

## 2 Methods

### 2.1 Study design

This study was conducted following the Preferred Reporting Items for Systematic Reviews and Meta-Analyses. The study protocol was registered in the International Prospective Register of Systematic Reviews (registration No.: CRD420251271276). This study adhered to PRISMA guidelines (14) and reported the required information accordingly.

### 2.2 Date source and search strategy

To comprehensively identify relevant studies, we systematically searched eight Chinese and English electronic databases from their inception to April 10, 2026. The English databases included PubMed, Embase, the Cochrane Library, and Web of Science, while the Chinese databases included China National Knowledge Infrastructure (CNKI), Wanfang Data, VIP Database, and the Chinese Biomedical Literature Database (CBM). No separate manual reference-list search or trial-registry search was performed beyond these eight prespecified bibliographic databases.

Search strategies were developed based on core concepts, including population, intervention, comparator, and study type, by combining controlled vocabulary terms with free-text terms. The key search terms were “cervical vertebrae,” “cervical radiculopathy,” “pulsed radiofrequency,” “PRF,” “RF,” “selective nerve root block,” “nerve block,” and “randomized controlled trial.” The complete search strategy for each database is provided in Supplementary material 1.

### 2.3 Inclusion criteria and study selection

#### 2.3.1 Types of studies

The inclusion criterion was restricted to randomized controlled trial (RCT), as strictly limiting the included studies to RCT can maximize internal validity, reduce the risk of bias, and meet the requirements of a high-quality meta-analysis.

#### 2.3.2 Types of participants

The study population comprised adults aged ≥18 years diagnosed with cervical radiculopathy or cervical nerve root pain according to the diagnostic definition of the North American Spine Society guideline (15). Cervical radiculopathy was defined as radicular pain in one or both upper extremities, with or without neck pain, related to compression or irritation of one or more cervical nerve roots (15). Eligible patients were required to have compatible clinical manifestations, such as neck–shoulder–arm pain, numbness, paresthesia, weakness, or reflex changes, together with MRI or CT evidence of corresponding nerve root compression, disc herniation, or foraminal stenosis at the clinically relevant level (15, 16).

#### 2.3.3 Types of intervention

The intervention in the experimental group consisted of pulsed radiofrequency applied to the cervical dorsal root ganglion or the affected nerve root, combined with nerve root block. The control group received nerve root block alone, with the type and dosage of injected medication kept consistent with those used in the experimental group. Interventions in both groups must be performed within the same treatment stage or clinical context to directly compare the therapeutic efficacy of “combined treatment” versus “injection therapy alone.”

#### 2.3.4 Types of measured outcomes

Eligible studies were required to report at least one of the following quantitative outcome measures: the primary outcome was pain intensity at 1, 3, and 6 months after treatment, assessed using either the visual analog scale or the numerical rating scale. Secondary outcomes included changes in Neck Disability Index scores to evaluate functional improvement, clinical efficacy rate, and the incidence of adverse events.

#### 2.3.5 Exclusion criteria

The exclusion criteria of the study were as follows: regarding study type, non-randomized controlled trials, cohort studies, case reports, reviews, and animal experiments were excluded. Regarding study participants, patients with concomitant cervical spondylotic myelopathy, spinal tumors, fractures, infections, or a history of previous cervical spine surgery were excluded. Regarding interventions, studies in which the interventions in the experimental and control groups were not conducted within the same treatment stage or clinical context were excluded, so as to ensure a direct comparison of the therapeutic effects between “combined therapy” and “injection therapy alone”.

## 3 Data extraction and analysis

### 3.1 Data extraction

The retrieved literature was first deduplicated using reference management software (EndNote), after which two researchers independently screened the titles and abstracts of the deduplicated records to exclude studies that were clearly irrelevant to the article’s content. Full-text screening was then performed according to the predefined inclusion and exclusion criteria. Two researchers independently conducted quality assessment and data extraction for the final included studies, followed by summary and cross-checking. During data extraction from the included articles, any discrepancies between the two researchers were reviewed and verified by a third independent researcher, and the final results were determined after discussion. The outcome indicators extracted from the studies included effective rate, incidence of adverse reactions, VAS score, cervical flexion-extension range of motion, Tanaka Yasuhisa cervical spondylosis symptom score, and related measures.

### 3.2 Quality assessment

The methodological quality of the included studies was assessed using the original Cochrane Risk of Bias (RoB) tool implemented in Review Manager version 5.4. The assessment covered the following seven domains: (1) implementation of randomization; (2) allocation concealment; (3) blinding of researchers and participants; (4) blinding of outcome assessors; (5) completeness of outcome data; (6) selective reporting; and (7) other potential sources of bias. The risk-of-bias assessment results were classified into three categories: high risk, low risk, or unclear risk.

### 3.3 Statistical analysis

This study performed the meta-analysis using RevMan 5.4 software. Effect sizes for continuous variables were expressed as mean differences (MDs) with 95% confidence intervals (CIs), with statistical significance defined as *p* < 0.05. Between-study heterogeneity was assessed using the I^2^ statistic: when *p* ≥ 0.1 and I^2^ < 50%, heterogeneity was considered minimal or absent, and a fixed-effects model was therefore applied. Conversely, when significant heterogeneity was present, a random-effects model was used. Because only a small number of studies contributed to the highly heterogeneous outcomes, formal subgroup analysis or meta-regression was not considered sufficiently robust. Potential sources of heterogeneity were therefore examined qualitatively according to follow-up time, baseline pain/function, PRF protocol, block technique and injectate, imaging guidance, clinical characteristics, and outcome measurement methods.

## 4 Results

### 4.1 Study search results

A total of 90 studies were retrieved according to the predefined search strategy. After removing duplicates, screening titles and abstracts, and reviewing the full texts, 9 studies that met the eligibility criteria were ultimately included (11, 17–24). Among them, 7 were Chinese-language studies, and 2 were English-language studies (11, 17). All included studies were randomized controlled trials. The literature screening process is shown in Figure 1.

**Figure 1:**
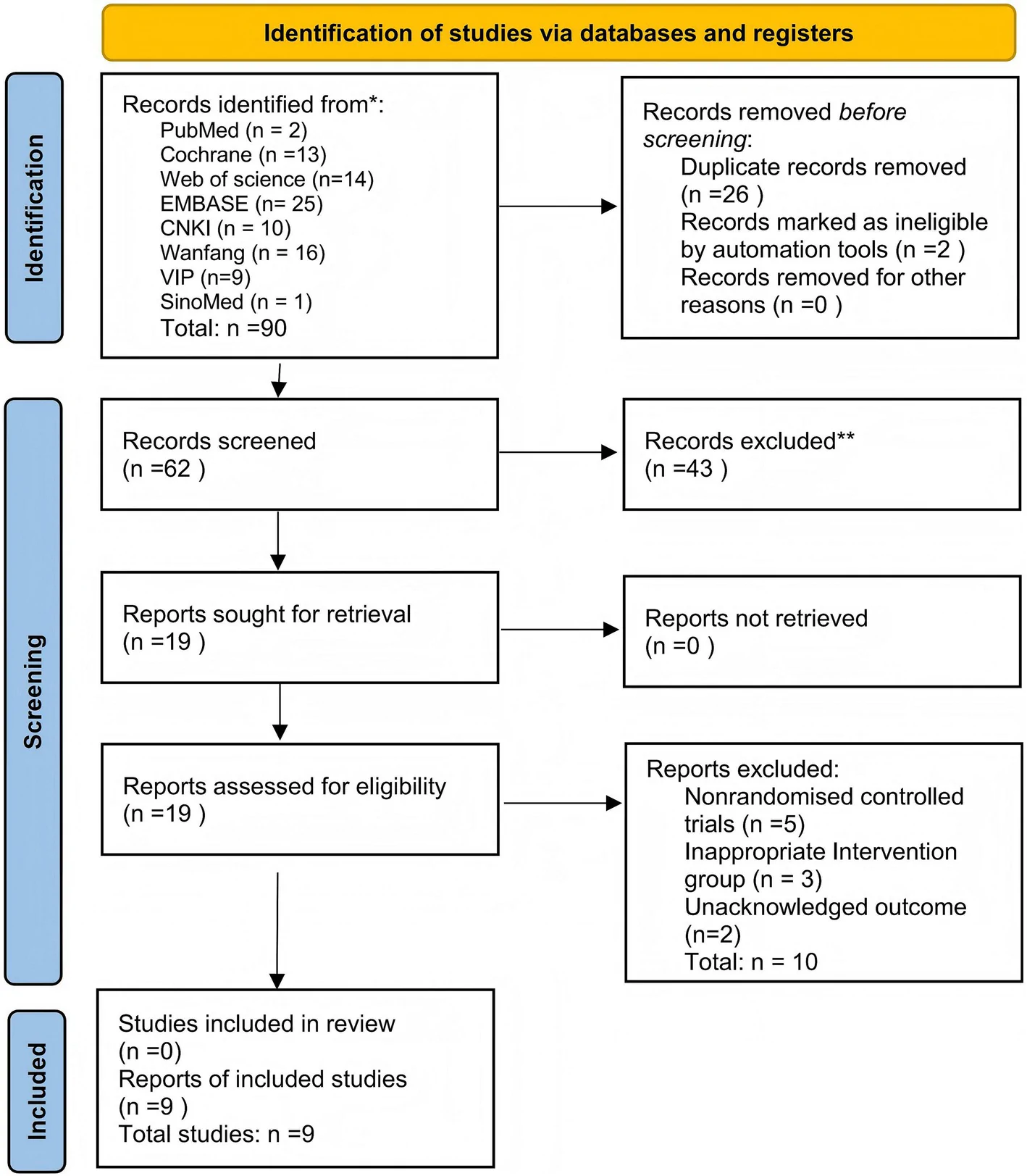
Flowchart of study selection.

### 4.2 Basic features of included studies

This study included a total of nine articles involving 783 patients. All included studies were randomized controlled trials.

#### 4.2.1 Intervention measures

All studies investigated the efficacy of pulsed radiofrequency combined with nerve block therapy for the treatment of cervical spondylotic radiculopathy. In the experimental groups, patients received pulsed radiofrequency combined with nerve block interventions: three studies used pulsed radiofrequency combined with foraminal injection or selective nerve root injection (18, 22, 24), three studies used pulsed radiofrequency combined with epidural block or nerve block (17, 20, 21, 23), one study used ultrasound-guided pulsed radiofrequency combined with steroid injection (11), and one study used pulsed radiofrequency combined with ultrasound-guided stellate ganglion block (19). The control groups received single-modality treatments, including nerve root injection, nerve block, epidural block, or stellate ganglion block. Most procedures were performed under ultrasound or fluoroscopic guidance, and the treatment frequency was generally either a single session or once weekly for 2 to 4 weeks. Treatment protocols were not uniform. PRF target/approach, imaging guidance, temperature, voltage or stimulation settings, pulse duration/frequency when available, treatment duration/cycles, and the nerve-block injectate/volume were extracted from the individual reports and are summarized in Table 1; items not reported by the original study are marked NR. These procedural differences are clinically plausible contributors to between-study heterogeneity.

**Table 1:** Summary of pulsed radiofrequency and comparator treatment parameters.

Study	PRF target/Approach	Guidance/Needle placement	PRF settings	Comparator intervention/Injectate	Schedule/Follow-up	Reporting note
Cui, 2025 (24)	PRF at the intervertebral foramen of the affected cervical level + intervertebral foraminal injection.	PRF: fluoroscopy (C-arm). Injection: surface-anatomy localization; no imaging guidance reported.	2 Hz; 42 °C; 120 s. For disease course >3 months: 5 Hz; 42 °C; 300 s. Pulse width/voltage/cycles: NR.	Intervertebral foraminal injection alone: 1 mL 2% lidocaine + 1 mL compound betamethasone + 3 mL 0.9% saline (total 5 mL).	Injection once weekly × 2 weeks; PRF single session. Exact timing of PRF relative to injections not specified.	Combination sequence not clearly reported.
Chen Q., 2025 (23)	Ultrasound-guided cervical nerve-root PRF + nerve block.	Ultrasound-guided.	NR.	Three-arm study with separate nerve-block and PRF comparator groups; drug and volume for the nerve-block arm were NR.	Once weekly × 2 weeks (2 sessions); follow-up at 1 month.	Three-arm design with separate nerve-block and PRF comparator groups.
Chen J., 2025 (22)	Selective cervical nerve-root block + PRF, both added to conventional medication.	Block: low-frequency ultrasound drug-delivery device/coupling patch at the target site. PRF: ultrasound-guided needle advanced near the nerve root.	2 Hz; pulse width 20 ms; 42 °C; 15 min; once weekly.	Received selective nerve-root block with 5% lidocaine + 0.5 mL compound betamethasone via an ultrasound drug-delivery patch; lidocaine volume NR.	Total course 1 month; weekly treatment (~4 sessions by frequency, but exact number not explicitly stated).	PRF was followed immediately by nerve-root block.
Tian, 2024 (21)	Cervical nerve-root PRF (C5-C7) + interlaminar epidural block.	Epidural: X-ray fluoroscopy at C6-7 or C7-T1 with catheter placement. PRF: ultrasound-guided; performed 3 days later.	2 Hz; pulse width 20 ms; 42 °C for 4 min + 45 °C for 4 min.	Epidural block alone: compound betamethasone 2 mg + Neurotropin 3 mL; epidural analgesia pump with vitamin B12 + lidocaine (amounts NR).	Single epidural block and single PRF; 3-day interval; follow-up at 1 week and 1, 3, and 6 months.	Three-arm study: epidural block, PRF, and combination.
Chalermkitpanit, 2023 (11)	Ultrasound-guided periradicular cervical nerve-root PRF + drug injection.	Ultrasound-guided.	NR.	Ultrasound-guided periradicular steroid injection; drug, dose, and volume NR.	Single treatment; follow-up at 1, 3, 6, and 9 months.	Detailed PRF and injectate parameters were not reported.
Yang, 2022 (20)	Bipolar PRF at the target intervertebral foramen/DRG region; monopolar PRF as a second active arm.	Ultrasound-guided placement; two RF needles positioned cranial/caudal to the target DRG region for bipolar PRF; CT used to confirm needle tips at the outer foramen. Monopolar arm used one needle with CT confirmation.	2 Hz; pulse width 20 ms; 42 °C; 240 s × 2 cycles (total 480 s); same parameters in the monopolar arm.	Epidural block at C7-T1 under ultrasound + CT: 1 mL 2% lidocaine + 1 mL compound betamethasone + mecobalamin 500 μg + 1 mL saline.	Single PRF session; epidural block appears single-session in the methods, but repetition was not explicitly stated.	Three-arm design comparing bipolar PRF, monopolar PRF, and epidural block.
Bai, 2022 (19)	Continuous PRF of the stellate ganglion + Stellate ganglion block	PRF: ultrasound-guided stellate ganglion target. Comparator block: no ultrasound guidance reported.	NR.	Stellate ganglion block; injectate/drug/volume NR.	Once weekly × 4 weeks (4 sessions).	The study compares continuous stellate-ganglion PRF with stellate-ganglion block.
Wang X., 2021 (18)	Dorsal root ganglion PRF + selective cervical nerve-root block.	C-arm fluoroscopy to the intervertebral foramen; block performed immediately after PRF. Comparator block also C-arm guided with contrast confirmation.	42 °C; 2 Hz; pulse width 20 ms; 120 s × 2 cycles per segment (total 240 s).	Selective nerve-root block alone: 3 mL anti-inflammatory/analgesic solution (1 mL 2% lidocaine + Diprospan 7 mg + 1 mL saline).	Single procedure; follow-up at 1 day, 7 days, 3 months, and 6 months.	PRF was followed immediately by nerve-root block.
Wang F., 2016 (17)	Posterior cervical nerve-root PRF + cervical nerve-root block (CNRB).	Fluoroscopy; posterior approach; 18G guide needle + 20G curved blunt RF cannula; target at the ‘U’-shaped depth of the transverse process. Position confirmed by sensory 50 Hz and motor 2 Hz stimulation (<0.6 V) plus 0.5–1 mL nonionic contrast.	Therapeutic PRF temperature, pulse width, duration, and number of cycles: NR. The 50 Hz/2 Hz and <0.6 V settings were used for stimulation and position confirmation rather than therapeutic PRF delivery.	CNRB alone: 5 mg betamethasone dipropionate + 2 mg betamethasone sodium phosphate + 1 mL 0.9% saline + 1 mL 2% lidocaine.	Single treatment; follow-up at 1 week and 1, 3, and 6 months.	Three-arm study: CNRB, PRF, and PRF + CNRB.

NR, not reported or not extractable from the included study.

#### 4.2.2 Outcome measures

All nine studies assessed pain intensity using either the visual analog scale or the numeric rating scale. Five studies evaluated cervical function using the Neck Disability Index or the Tanaka Yasuhisa Cervical Spondylosis Symptom Scale (11, 17, 18, 21, 23). Three studies reported cervical range of motion (19, 22, 24). For cervical flexion–extension range of motion, the included reports did not consistently describe the measurement instrument, patient positioning, or standardized measurement protocol; therefore, measurement standardization across studies could not be confirmed. Two studies measured inflammatory markers, including interleukin-6 and tumor necrosis factor-α (20, 24). Four studies used the Odom criteria to assess overall clinical efficacy (17, 18, 21, 24). All studies recorded complications and adverse events. To clearly present differences among the included studies in terms of sample size, age, intervention protocols, and outcome measures, a summary table is provided (Table 2).

**Table 2:** Characteristics of included studies.

Included studies	Sample size	Age (years old)		Intervention measure		Course of treatment	Efficacy indicator
	T/C	T	C	Treatment group	Control group		
Cui, 2025 (24)	59/59	52.16 ± 11.14	52.51 ± 9.94	PRF + NRB	NRB	2 weeks (once a week)	ABCDFG
Chen, 2025 (23)	35/35	49.51 ± 9.55	49.63 ± 9.02	PRF + NRB	NRB	4 weeks (once a week)	CD
Chen, 2025 (22)	50/50	53.94 ± 12.55	54.32 ± 13.73	PRF + NRB	NRB	2 weeks (once a week)	ABCE
Tian, 2024 (21)	58/60	59.14 ± 10.78	59.07 ± 9.70	PRF + NRB	NRB	Single therapy	ABCH
Chalermkitpanit, 2023 (11)	20/21	48.9 ± 15.8	56.3 ± 14.6	PRF + NRB	NRB	Single therapy	HIJK
Yang, 2022 (20)	40/40	63.2 ± 6.8	63.8 ± 6.6	PRF + NRB	NRB	Single therapy	AILM
Bai, 2022 (19)	35/35	45.97 ± 5.42	45.24 ± 5.92	PRF + NRB	NRB	4 weeks (once a week)	ENO
Wang, 2021 (18)	36/36	49.2 ± 8.1	48.5 ± 9.2	PRF + NRB	NRB	Single therapy	AC
Wang, 2016 (17)	21/21	57.5 ± 15.0	59.0 ± 13.8	PRF + NRB	NRB	Single therapy	IJ

T, treatment group; C, control group; PRF, pulsed radiofrequency; NRB, nerve block; VAS, Visual analog scale; A, Efficiency analysis; B, Incidence of adverse reactions; C, VAS score; D, Cervical flexion and extension activity; E, Yasuyuki Tanaka Cervical Spondylosis Symptom Scale; F, IL-6; G, TNF-α; H, NDI score of cervical spine function; I, Pain degree score; NRS; J, pain duration; K, The maintenance time of patients with 50% pain relief after intervention; L, JOA score; M, Temperature difference of infrared thermal imaging of posterior bilateral upper limbs; N, The level of vertebral artery blood flow index; O, Comparison of sympathetic symptom score.

### 4.3 Quality assessment

The risk of bias of the included studies was assessed using the Cochrane Risk of Bias tool, with judgments of low, high, or unclear risk made across seven domains: random sequence generation, allocation concealment, blinding of participants and personnel, blinding of outcome assessors, completeness of outcome data, selective reporting, and other sources of bias. Random sequence generation: all nine RCTs (11, 17–24) were randomized controlled trials; and all nine studies (11, 17–24) used a random number table. Allocation concealment: all nine studies (11, 17–24) reported complete outcome data and were rated as having a low risk of bias. Performance bias: four studies (11, 18, 22, 24) reported blinding of participants and were rated as having a low risk of bias, whereas the remaining five studies (17, 19–21, 23) did not provide relevant information and were therefore rated as having an unclear risk of bias. Detection bias: four studies (11, 17, 22, 24) reported blinding of outcome assessors and were rated as having a low risk of bias, whereas the remaining five studies (18–21, 23) did not report this information and were therefore rated as having an unclear risk of bias. Attrition bias: six studies (11, 19, 20, 22–24) reported complete outcome data and were rated as having a low risk of bias, one study (21) did not report this information and were therefore rated as having an unclear risk of bias, whereas two studies (17, 18) had missing data and were rated as having a high risk of bias. Reporting bias: all nine studies (11, 17–24) reported complete outcome data and were rated as having a low risk of bias. Other bias: all nine studies (11, 17–24) reported complete outcome data and were rated as having a low risk of bias, as shown in Figures 2a,b.

**Figure 2:**
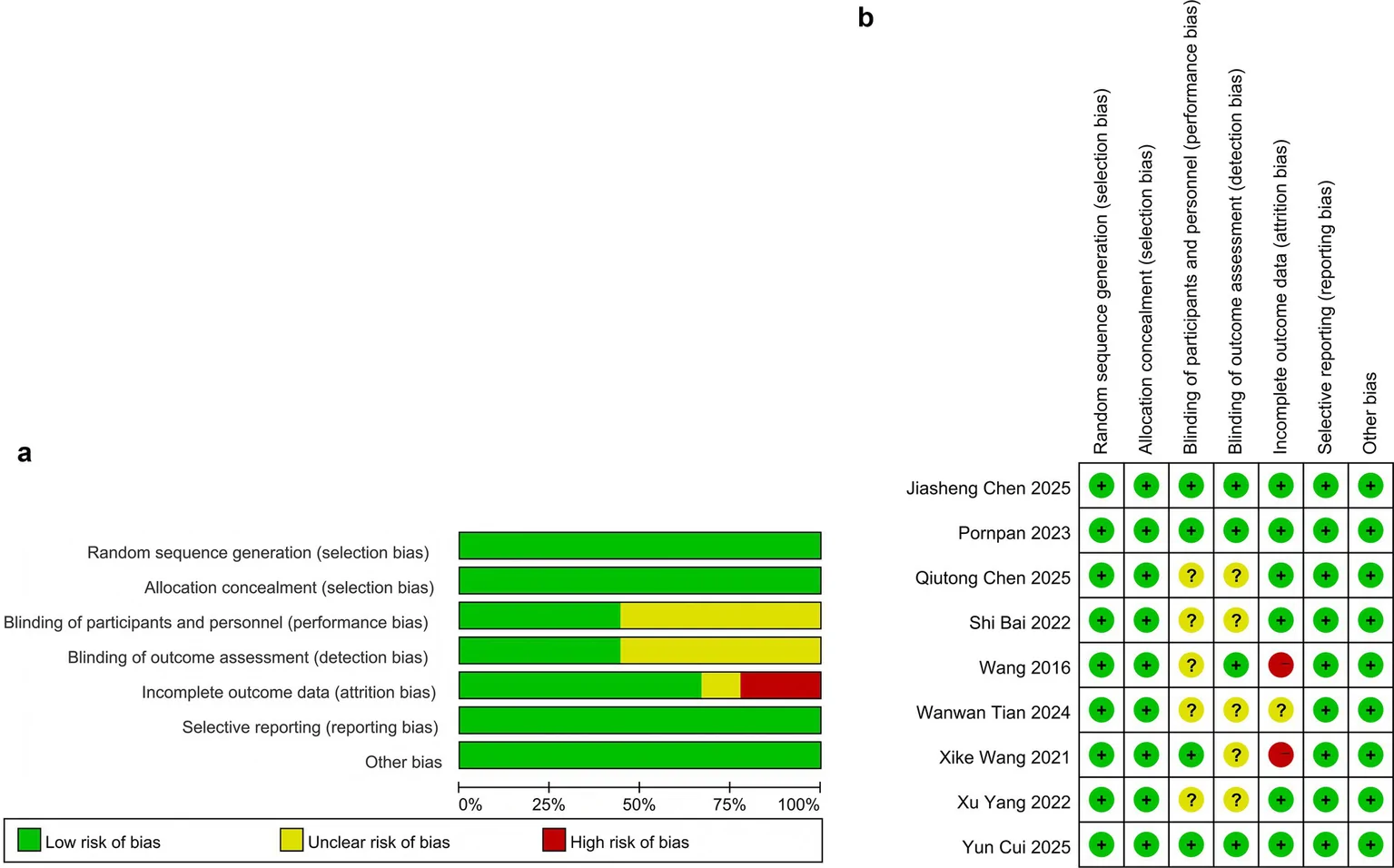
Cochrane bias risk assessment results for included studies **(a,b)**.

### 4.4 Meta analysis result

#### 4.4.1 Overall effective rate

Five included studies (18, 20, 21, 23, 24) reported the total effective rate, involving 480 patients in total, including 240 patients in the pulsed radiofrequency combined with nerve block group and 240 patients in the nerve block group. As shown in Figure 3, the heterogeneity test yielded *p* = 0.27 and I^2^ = 23%, indicating low heterogeneity among the included studies; therefore, a fixed-effect model was used for the systematic evaluation. The pooled analysis showed that the overall risk ratio (RR) for the effective rate was 1.13 (95% confidence interval [CI]: 1.05–1.22), with a Z value of 3.32 (*p* = 0.0009), suggesting that the combined therapy may have greater advantages in improving the effective rate for cervical radiculopathy.

**Figure 3:**
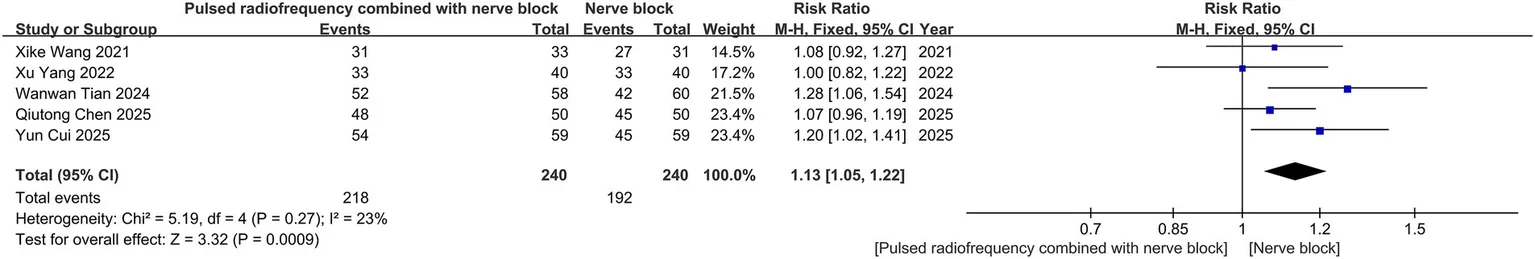
Forest plot of the overall effective rate.

#### 4.4.2 Incidence of adverse reactions

A total of three studies (21, 23, 24) reported the incidence of adverse reactions after intervention, involving 336 patients, including 167 in the pulsed radiofrequency combined with nerve block group and 169 in the nerve block group. As shown in Figure 4, the heterogeneity test yielded *p* = 0.26 and I^2^ = 26%, indicating low heterogeneity among the included studies; therefore, a fixed-effects model was used for the systematic review. The analysis showed that the pooled risk ratio (RR) for the incidence of adverse reactions was 0.96 (95% confidence interval [CI]: 0.52–1.78), with a Z value of 0.12 (*p* = 0.91), indicating no statistically significant difference in the incidence of adverse reactions between the two groups.

**Figure 4:**
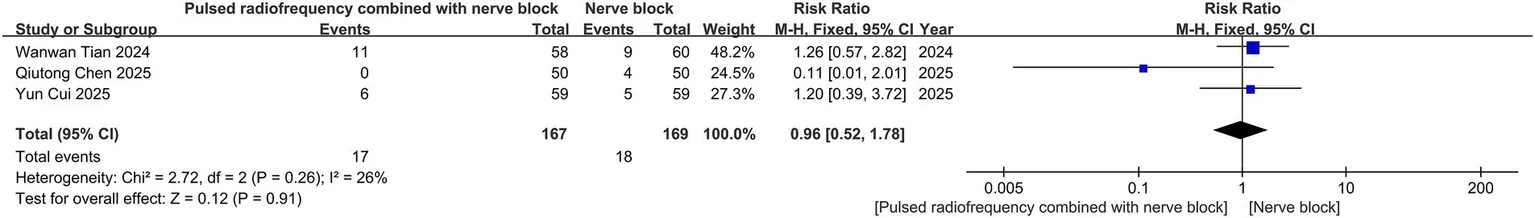
Forest plot of the incidence of adverse reactions.

#### 4.4.3 VAS score

A total of five studies (18, 21–24) reported post-intervention VAS scores, involving 470 patients, including 235 patients in the pulsed radiofrequency combined with nerve block group and 235 patients in the nerve block group. As shown in Figure 5, the heterogeneity test yielded *p* < 0.00001 and I^2^ = 89%, indicating substantial heterogeneity among the included studies, which may be related to differences in follow-up time points, baseline scores, or assessment tools across studies; therefore, a random-effects model was used for the systematic evaluation. The analysis showed that the mean difference was −0.87 (95% confidence interval [CI]: −1.27 to −0.47), with a Z value of 4.23 (*p* < 0.0001), indicating that the combined therapy produced more pronounced improvement in clinical symptoms. Although considerable heterogeneity was observed among the included studies (I^2^ = 89%), all included studies suggested that combined treatment was superior to nerve block alone in improving clinical symptoms. Because only five studies contributed to this outcome, formal subgroup analyses would be unstable. The heterogeneity may reflect differences in follow-up time points, baseline pain severity, pain-scale implementation, PRF parameters, nerve-block targets and injectates, imaging guidance, symptom chronicity, etiology (e.g., disc herniation versus foraminal stenosis), prior treatments, and patient selection. Accordingly, the pooled pain effect should be interpreted as an average effect across clinically heterogeneous protocols rather than as a uniform treatment effect.

**Figure 5:**
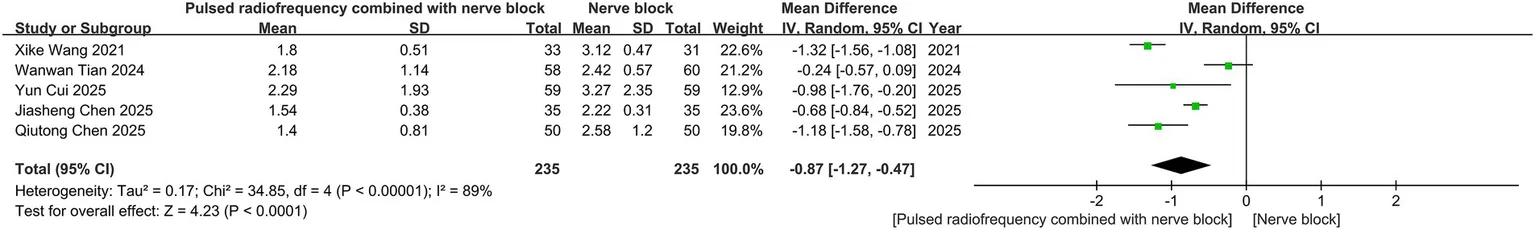
Forest plot of VAS scores.

#### 4.4.4 Cervical flexion–extension range of motion

A total of two studies (22, 24) reported post-intervention cervical flexion–extension range of motion, including 188 patients, with 94 patients in the pulsed radiofrequency combined with nerve block group and 94 patients in the nerve block group. As shown in Figure 6, the heterogeneity test yielded *p* = 0.0003 and I^2^ = 92%, indicating substantial heterogeneity among the included studies. This heterogeneity may be related to differences in baseline cervical function, disease duration, degree of initial restriction in cervical motion, PRF parameter settings, nerve block targets, follow-up duration, and the standardization of range-of-motion assessment. Therefore, a random-effects model was used for the meta-analysis. The pooled result showed that the mean difference was 8.56 (95% CI: 1.67, 15.44), with a Z value of 2.43 (*p* = 0.01), suggesting that PRF combined with nerve block may be associated with greater improvement in cervical flexion–extension range of motion than nerve block alone. However, this finding should be interpreted cautiously because it was based on only two studies and showed considerable between-study heterogeneity. Further well-designed trials with standardized measurement methods and longer follow-up are needed to verify the reliability and clinical significance of this functional benefit. Because the two pooled studies did not consistently report identical range-of-motion instruments or measurement procedures, part of the observed heterogeneity may be measurement-related.

**Figure 6:**
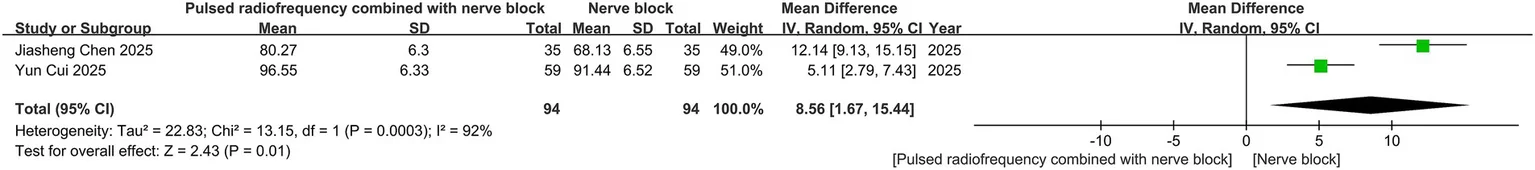
Forest plot of cervical flexion-extension range of motion.

#### 4.4.5 Yasuhisa Tanaka score

Two studies (19, 23) reported post-intervention Yasuhisa Tanaka scores, involving 164 patients in total, including 83 patients in the pulsed radiofrequency combined with nerve block group and 81 patients in the nerve block group. As shown in Figure 7, the heterogeneity test yielded *p* = 0.31 and I^2^ = 3%, indicating low heterogeneity among the included studies; therefore, a fixed-effect model was used for the systematic review. The analysis showed that the mean difference was 2.61 (95% confidence interval [CI]: 1.81–3.40), with a Z value of 6.40 (*p* < 0.00001), indicating that improvement in the Yasuhisa Tanaka score was significantly greater in the pulsed radiofrequency combined with nerve block group than in the nerve block alone group.

**Figure 7:**
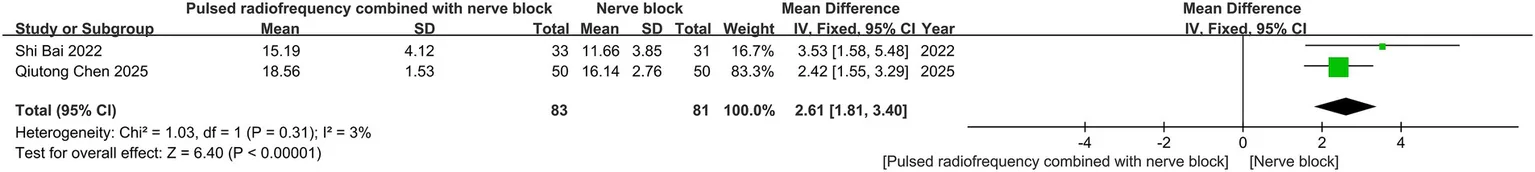
Forest plot of the Yasuhisa Tanaka score.

### 4.5 Pooled outcomes

As shown in Table 3, the meta-analysis demonstrated that pulsed radiofrequency combined with nerve block was associated with a significantly higher overall treatment efficiency than the control intervention, with low heterogeneity among the included studies (5 studies, 480 participants; RR = 1.13, 95% CI: 1.05–1.22; I^2^ = 23%; *p* = 0.0009). Regarding safety, no significant difference was observed in the incidence of adverse reactions between the two groups (3 studies, 336 participants; RR = 0.96, 95% CI: 0.52–1.78; I^2^ = 26%; *p* = 0.91). For pain relief, the combined treatment significantly reduced VAS scores compared with the control treatment, although substantial heterogeneity was detected (5 studies, 470 participants; MD = −0.87, 95% CI: −1.27 to −0.47; I^2^ = 89%; *p* < 0.0001). Functional outcomes also favored the combined intervention, as reflected by improved cervical flexion and extension activity (2 studies, 188 participants; MD = 8.56, 95% CI: 1.67–15.44; I^2^ = 92%; *p* = 0.01) and higher Yasuyuki Tanaka Cervical Spondylosis Symptom Scale scores (2 studies, 164 participants; MD = 2.61, 95% CI: 1.81–3.40; I^2^ = 3%; *p* < 0.00001).

**Table 3:** Pooled outcomes of pulsed radiofrequency combined with nerve block for cervical radiculopathy.

Outcome measurements	Included studies	Participants	I^2^	Statistic effect model	Effect estimate	*p*-value
Efficiency analysis	5	480	23%	RR (M-H, Fixed, 95% CI)	1.13 [1.05, 1.22]	0.0009
Incidence of adverse reactions	3	336	26%	RR (M-H, Fixed, 95% CI)	0.96 [0.52, 1.78]	0.91
VAS score	5	470	89%	MD (IV, Random, 95% Cl)	−0.87 [−1.27,-0.47]	*p* < 0.0001
Cervical flexion and extension activity	2	188	92%	MD (IV, Random, 95% Cl)	8.56 [1.67, 15.44]	0.01
Yasuyuki Tanaka Cervical Spondylosis Symptom Scale	2	164	3%	MD (IV, Fixed,95% CI)	2.61 [1.81, 3.40]	*p* < 0.00001

VAS, visual analog scale; CI, confidence interval; RR, risk ratio; MD, mean difference.

### 4.6 Assessment of publication bias

Potential publication bias for the response rate outcome was assessed by visual inspection of the funnel plot and Egger’s regression test. As shown in Figure 8, the funnel plot suggested possible asymmetry, with fewer studies located in the lower-left region. However, because only five studies were included, visual assessment of funnel plot symmetry is inherently uncertain, and the apparent asymmetry may reflect chance, between-study heterogeneity, small-study effects, or publication bias.

**Figure 8:**
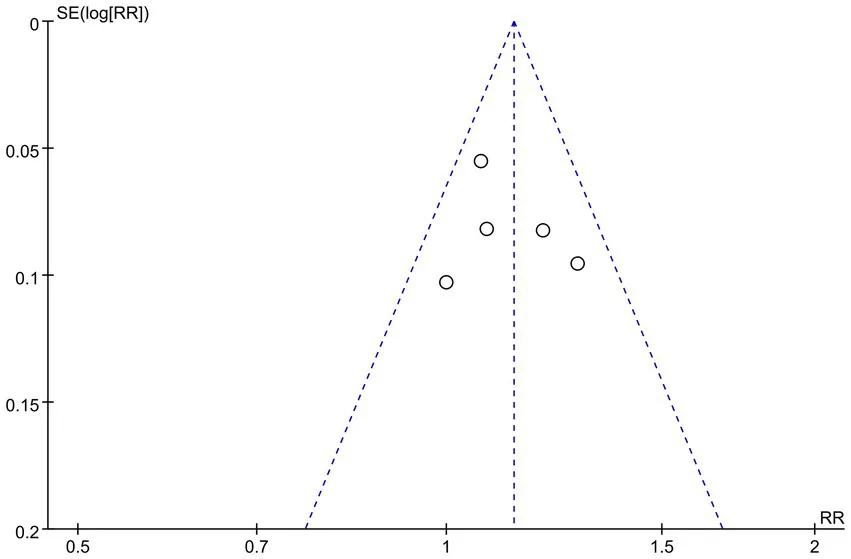
Funnel plot of the total effective rate.

Egger’s regression test yielded β₁ = 1.21 (*p* = 0.612), indicating no statistically significant evidence of funnel plot asymmetry. Nevertheless, given the limited statistical power of Egger’s test when the number of included studies is small, the non-significant result should not be interpreted as evidence that publication bias is absent. Therefore, publication bias cannot be reliably excluded, and the results of this assessment should be interpreted with caution.

## 5 Discussion

This study systematically evaluated the clinical value of pulsed radiofrequency combined with nerve block for the treatment of cervical spondylotic radiculopathy. The results showed that, compared with nerve block alone, the combined treatment was superior in improving the overall clinical response rate, reducing pain intensity, enhancing cervical flexion–extension range of motion, and improving the Tanaka Yasuhisa cervical spondylosis symptom score, without increasing the incidence of adverse reactions. These findings indicate a possible incremental benefit of combination therapy during the reported follow-up periods, but they do not establish a sustained therapeutic effect because longitudinal data were limited and follow-up time points were not pooled consistently.

The pathological basis of cervical spondylotic radiculopathy is not limited to mechanical compression, but also involves nerve root edema, release of inflammatory mediators, abnormal discharge of the dorsal root ganglion, and central sensitization (25, 26). Nerve block alone mainly achieves rapid analgesia by reducing local inflammation and blocking peripheral nociceptive afferent input through local anesthetics and glucocorticoids. However, the duration of its effect is constrained by drug metabolism and recurrence of local inflammation (27, 28). Pulsed radiofrequency acts on the nerve root or dorsal root ganglion within a temperature range that does not cause thermal coagulative destruction, and can regulate abnormal neural discharge, neuroimmune responses, and inflammatory factor expression (10, 29, 30). Therefore, the advantage of combined treatment may be explained by the complementary effect between “rapid anti-inflammatory analgesia” and “sustained neuromodulation,” which is also consistent with the simultaneous improvement in VAS scores and function-related indicators observed in this study. The pooled pain effect (MD = −0.87 points) was statistically significant, but its clinical importance is uncertain. In cervical radiculopathy, an MCID of 2.2 points has been reported for a 0–10 Numeric Pain Rating Scale (31). Although this value is not directly interchangeable with VAS, it underscores that statistical significance should not automatically be interpreted as a clinically meaningful between-group benefit.

The findings of this study regarding pain improvement are consistent with the direction of previous high-quality evidence. The double-blind, sham-controlled randomized trial by Van Zundert et al. confirmed that pulsed radiofrequency targeting the cervical dorsal root ganglion produced greater pain relief than sham treatment in patients with chronic cervical radicular pain (8). The meta-analysis by Kwak et al. further showed that pulsed radiofrequency could continuously reduce pain scores at follow-ups of 2 weeks, 1 month, 3 months, and 6 months (10). A recent randomized controlled study by Chalermkitpanit et al., using ultrasound-guided pulsed radiofrequency of the cervical nerve root, found that pulsed radiofrequency prolonged pain relief and improved neck function compared with steroid injection alone (11). Unlike these studies, the present study focused on a direct comparison between “pulsed radiofrequency combined with nerve block” and “nerve block alone,” and integrated multidimensional outcomes including overall response rate, pain, cervical range of motion, and symptom scores; therefore, it more closely reflects real-world treatment pathways in clinical decision-making. Because cervical range of motion and Yasuhisa Tanaka scores were each informed by only two studies, these findings should be viewed as exploratory evidence of possible functional benefit rather than definitive proof of clinically meaningful functional recovery.

Improvement in functional outcomes is an important finding of this study. Pain relief itself does not necessarily translate into improved cervical range of motion and daily function, whereas the increases in cervical flexion–extension range of motion and Tanaka Yasuhisa score observed in this study indicate that combined treatment has functional significance in alleviating nerve root irritation and protective muscle spasm. Previous studies have shown that cervical radicular pain is often accompanied by neck, shoulder, and arm pain, sensory abnormalities, and restricted movement, and that its diagnostic and therapeutic goals should cover both pain control and functional recovery (32–34). Kinci et al. also observed mid-term improvements in pain and patient satisfaction in a study of pulsed radiofrequency combined with cervical epidural steroid injection (35). This study further supports that, in patients with insufficient response to conservative treatment and without clear surgical indications, a combined minimally invasive interventional strategy may serve as a reasonable option within stepwise treatment.

In terms of safety, this study showed that the combined treatment did not increase the incidence of adverse reactions. However, this result should be interpreted as “no increase in common short-term adverse reactions,” rather than as sufficient exclusion of rare serious complications. The cervical region has complex anatomy, with close proximity among the nerve roots, vertebral artery, and dural sac; therefore, any transforaminal or selective nerve root procedure must strictly rely on imaging guidance and standardized techniques. Jee et al. indicated that ultrasound guidance may offer advantages in real-time visualization of neurovascular structures (6). The safety study by Beckworth et al. also emphasized the importance of nonparticulate glucocorticoids and standardized procedures in reducing catastrophic complications (13). Most procedures in the studies included in this review were performed under ultrasound or fluoroscopic guidance, which provides a reasonable explanation for the absence of additional observed safety risks; nevertheless, future studies should still uniformly record vascular puncture, transient neuritis, paresthesia, infection, and long-term neurological outcomes.

The academic significance of this study lies in advancing the existing evidence, which has mainly focused on pulsed radiofrequency alone or injection alone, to the level of systematic evaluation of combined treatment strategies. Current evidence shows that epidural or selective nerve root injections can improve short-term symptoms in some patients with cervical radicular pain, but uncertainties remain regarding long-term benefits and the need for repeated treatment (4, 6, 36, 37). The present pooled data suggest that adding pulsed radiofrequency may provide additional analgesic and functional benefit during the observed follow-up intervals, but they do not establish that this benefit is durable over the long term. Its potential application value lies in providing a minimally invasive option between medication/rehabilitation and surgery for patients with refractory cervical spondylotic radiculopathy, while also offering evidence for the future development of individualized interventional treatment protocols.

This study still has several limitations. First, the number of included studies and sample size were limited, and some outcomes were contributed by only 2–5 studies, which restricted the precision of the effect estimates. Second, VAS scores and cervical flexion–extension range of motion showed relatively high heterogeneity, which may have arisen from differences in follow-up duration, pulsed radiofrequency parameters, nerve block targets, injected drugs, imaging guidance methods, and baseline disease course. Third, some studies did not adequately describe allocation concealment, participant blinding, or outcome assessor blinding, which may have overestimated the treatment effect. Fourth, existing studies have insufficiently reported long-term recurrence rates, the need for repeated interventions, reductions in analgesic use, conversion to surgery, and minimal clinically important differences, thereby limiting extrapolation of the results to long-term clinical benefit. Clinical heterogeneity also warrants caution because symptom duration, etiology (disc herniation versus foraminal stenosis), chronicity, prior conservative/interventional treatments, and patient-selection criteria varied or were incompletely reported across trials. Seven of the nine included RCTs were conducted in China; regional differences in interventional practice patterns, patient selection, injectates, imaging guidance, and outcome conventions may therefore limit external validity to other healthcare settings. Possible language and publication bias cannot be excluded, particularly because the search did not include trial registries or a separate hand-search beyond the prespecified databases. In addition, heterogeneous PRF protocols and non-standardized outcomes such as overall clinical response rate and the Yasuhisa Tanaka score reduce cross-study comparability, and no formal GRADE assessment was performed to rate the certainty of evidence by outcome. Future studies should conduct multicenter, large-sample, double-blind randomized controlled trials, with unified pulsed radiofrequency parameters, injection protocols, follow-up time points, and core outcome sets prespecified, while also incorporating subgroup analyses based on inflammatory factors, neurological function assessments, and imaging characteristics.

Overall, this study supports that pulsed radiofrequency combined with nerve block can improve pain and functional outcomes in patients with cervical spondylotic radiculopathy without increasing short-term adverse reactions. However, the pooled pain effect was modest and may not meet established clinically important thresholds, while long-term durability and international generalizability remain uncertain. Future research should move beyond the question of “whether it is effective” toward three key questions: “which patients benefit the most, what parameters are optimal, and whether the therapeutic effect can be maintained over the long term,” so as to promote the more standardized and precise clinical application of this combined strategy.

## Data Availability

The original contributions presented in the study are included in the article/Supplementary material, further inquiries can be directed to the corresponding authors.
