# Supplementary material for: Efficacy and safety of pulsed radiofrequency combined with nerve block for cervical radiculopathy: a systematic review and meta-analysis
Source: Front Neurol. 2026 Sep 18;17:1877201. doi: 10.3389/fneur.2026.1877201 (PMC13630689; doi:10.3389/fneur.2026.1877201)
Supplement: Supplementary file 1 [file Supplementary_file_1.docx]

Supplementary Material

# 1 Search strategies

**1.1 PubMed: 2 records.**

| #1 | ((("cervical vertebrae"[MeSH Terms] OR "cervical spine"[Title/Abstract] OR "cervical radiculopathy"[Title/Abstract] OR "cervical radicular pain"[Title/Abstract] OR "cervical disc herniation"[Title/Abstract])AND("pulsed radiofrequency"[Title/Abstract] OR "pulsed RF"[Title/Abstract] OR "PRF"[Title/Abstract])AND("nerve block"[MeSH Terms] OR "nerve block"[Title/Abstract] OR "nerve blocks"[Title/Abstract] OR "neural blockade"[Title/Abstract] OR "epidural steroid injection"[Title/Abstract] OR "selective nerve root block"[Title/Abstract]))) NOT ("review"[Publication Type] OR "systematic review"[Publication Type] OR "meta-analysis"[Publication Type] OR "animals"[MeSH Terms] NOT "humans"[MeSH Terms]) |
| --- | --- |

**1.2 The Cochrane library: 13 records.**

| #1 | MeSH descriptor: [Cervical Spondylotic Radiculopathy] explode all trees |
| --- | --- |
| #2 | (cervical radiculopathy OR cervical radicular pain):ti,ab,kw |
| #3 | (pulsed radiofrequency OR pulsed RF OR PRF):ti,ab,kw |
| #4 | (nerve root block OR selective nerve root block OR transforaminal injection):ti,ab,kw |
| #5 | #1 OR #2 AND #3 AND #4 |
| #6 | Publication Type: "Trial" OR "Randomized Controlled Trial" |
| #7 | #5 AND #6 |

**1.3 Web of science: 14 records.**

| #1 | TS=("cervical spondylotic radiculopathy" OR "cervical radiculopathy" OR "cervical radicular pain") |
| --- | --- |
| #2 | TS=("pulsed radiofrequency" OR "pulsed RF" OR "PRF") |
| #3 | TS=("nerve root block" OR "selective nerve root block" OR "transforaminal injection") |
| #4 | TS=("randomized controlled trial" OR "randomised" OR "randomized" OR "RCT") |
| #5 | #1 AND #2 AND #3 AND #4 |

**1.4 EMBASE: 15 records.**

| #1 | exp cervical spondylotic radiculopathy/ or (cervical spondylotic radiculopathy or cervical radiculopathy or cervical radicular pain or cervical root pain or cervical nerve root compression).ti,ab,kw. |
| --- | --- |
| #2 | exp pulsed radiofrequency/ or (pulsed radiofrequency or PRF or pulsed RF).ti,ab,kw. |
| #3 | exp nerve root block/ or (nerve root block* or selective nerve root block* or transforaminal epidural steroid injection* or nerve root injection).ti,ab,kw. |
| #5 | #1 and #4 |
| #6 | exp randomized controlled trial/ or random*.ti,ab,kw. or controlled clinical trial/ or placebo.ti,ab,kw. |
| #7 | #5 and #6 |

**1.5 CNKI: 10 records.**

| #1 | 主题：颈椎病 + 神经根型颈椎病 + 颈神经根病 + 颈神经根性疼痛 |
| --- | --- |
| #2 | 主题：脉冲射频 + PRF |
| #3 | 主题：神经根阻滞 + 选择性神经根阻滞 + 椎间孔注射 + 硬膜外类固醇注射 |
| #4 | 篇关摘：随机 + 随机对照 + RCT |
| #5 | #1 AND #2 AND #3 AND #4 |

**1.6 Wanfang: 16 records.**

| #1 | 题目或关键词=(神经根型颈椎病 OR 颈神经根病 OR 颈神经根性疼痛) |
| --- | --- |
| #2 | 题目或关键词=(脉冲射频 OR PRF) |
| #3 | 题目或关键词=(神经根阻滞 OR 选择性神经根阻滞 OR 椎间孔注射) |
| #4 | 题目或关键词=(随机对照 OR 随机 OR RCT) |
| #5 | #1 AND #2 AND #3 AND #4 |

**1.7 VIP: 9 records.**

| #1 | 题目或关键词=(颈椎病 + 神经根型颈椎病 + 颈神经根病)) |
| --- | --- |
| #2 | 题目或关键词=(脉冲射频 + PRF)) |
| #3 | 题目或关键词=(神经根阻滞 + 选择性神经根阻滞 + 椎间孔注射) |
| #4 | 题目或关键词=(随机对照试验 + 随机 + RCT) |
| #5 | #1 AND #2 AND #3 AND #4 |

**1.8 SinoMed: 1 records.**

| #1 | "颈椎病"[常用字段:智能] OR "神经根型颈椎病"[常用字段:智能] |
| --- | --- |
| #2 | "射频消融"[常用字段:智能] OR "PRF"[常用字段:智能] |
| #3 | "神经根阻滞"[常用字段:智能] OR "椎间孔注射"[常用字段:智能] |
| #4 | "随机对照试验"[常用字段:智能] OR "随机"[常用字段:智能] OR "RCT"[常用字段:智能] |
| #5 | #1 AND #2 AND #3 AND #4 |
